# Placental Mesenchymal Stromal Cells (PMSCs) and PMSC-Derived Extracellular Vesicles (PMSC-EVs) Attenuated Renal Fibrosis in Rats with Unilateral Ureteral Obstruction (UUO) by Regulating CD4^+^ T Cell Polarization

**DOI:** 10.1155/2020/2685820

**Published:** 2020-07-22

**Authors:** Zhu Zhu, Chaonan Han, Shuli Xian, Feng Zhuang, Feng Ding, Wei Zhang, Yingli Liu

**Affiliations:** Department of Nephrology, Shanghai Ninth People's Hospital, Shanghai Jiao Tong University School of Medicine, Shanghai 200011, China

## Abstract

**Purpose:**

Recent evidence has shown that CD4^+^ T helper (Th) cells are involved in renal inflammation and fibrosis. However, whether renal fibrosis can be alleviated by intervening in the polarization of CD4^+^ T cells remains unknown. Our research investigated the effects of intravenously administered placenta mesenchymal stromal cells (PMSCs) or treatment with extracellular EVs (EVs) derived from PMSCs (PMSC-EVs) on the polarization of CD4^+^ T cells in rats with unilateral ureteral obstruction (UUO). We further verified how PMSCs affect inflammatory factor secretion and the levels of regulatory T (Treg) and Th17 CD4^+^ T cells in vitro.

**Materials and Methods:**

We evaluated renal interstitial inflammation and fibrosis by pathological section staining, tested the polarization of CD4^+^ T cells (Th17 and Treg phenotypes) by flow cytometry (FCM) and immunohistochemistry, and detected the cytokines secreted by CD4^+^ T cells by enzyme-linked immunosorbent assay (ELISA).

**Results:**

Compared with that of control rats, the renal tissue of PMSC-treated rats exhibited lower renal Masson scores and more Foxp3^+^ cell infiltration, with a significantly decreased IL17A^+^CD4^+^ T cell/CD4^+^ T cell ratio and a significantly elevated anti-inflammatory cytokine (IL-10) level. When CD4^+^ T cells were cocultured with PMSCs, CD4^+^IL17A^+^ cell percentages were decreased in a UUO model after 7 days of coculture with PMSCs. The secretion of TGF-*β* and IL-10 was significantly increased (*P* < 0.05), while the secretion of IFN-*γ*, IL-17, and IL-6 was significantly decreased (*P* < 0.05) in the PMSC coculture group. Moreover, after treatment with PMSC-EVs, tubulointerstitial fibrosis was alleviated, and Foxp3^+^/IL-17^+^ cell infiltration was increased in the kidneys of UUO model animals on day 7.

**Conclusions:**

PMSCs can convert the inflammatory environment into an anti-inflammatory environment by affecting the polarization of CD4^+^ T cells and macrophages, inhibiting the inflammatory factors IFN-*γ* and IL-17, and upregulating the expression of the anti-inflammatory factors TGF-*β* and IL-10, ultimately leading to renal protection. Such functions may be mediated by the paracrine activity of PMSC-EVs.

## 1. Introduction

Unilateral ureteral obstruction- (UUO-) induced subacute renal injury is characterized by tubular cell injury, interstitial inflammation, and renal fibrosis. Recent studies indicated that intervening in the polarization of CD4^+^ T cells could be a potential therapeutic approach to prevent excessive fibrosis and loss of renal function due to injury [1–3]. Based on their cytokine secretion profiles and the expression of specific transcription factors, CD4^+^ T cells are classified into four major subpopulations: T helper (Th) 1, Th2, Th17, and regulatory T (Treg) cells, and additional Th cell lineages might exist. IFN-*γ* is known to induce Th1 cell production, and interleukin (IL)-17 increases CD4^+^ T cell proliferation that differentiates naïve CD4^+^ T cells into Th17 cells, which are considered proinflammatory T cells. Treg cells and IL-4-induced Th2 cells are identified as anti-inflammatory subsets [4–6]. These studies indicate that the polarization of immune cells is vital to maintaining homeostasis and inflammatory processes.

Mesenchymal stromal cells (MSCs) are multipotent stromal cells characterized by their abilities to differentiate into cells that compose mesodermal tissue and inhibit the proliferation of T and B lymphocytes, natural killer cells, and dendritic cells both in vitro and in vivo, making them an effective stromal cell source for regenerative medicine [7]. Nevertheless, the application of MSCs derived from the bone marrow (BM-MSCs) has essential limitations, including the invasive harvest procedure and limited accessibility due to the low cell yield [8, 9].

Our previous study found that BM-MSCs transformed an inflammatory environment into an anti-inflammatory environment to induce immune tolerance by inhibiting the inflammatory factor IFN-*γ*, upregulating the expression of the anti-inflammatory factor IL-10, and regulating the Treg cell population and function [10]. In this study, we isolated MSCs from the placenta (PMSCs); this procedure was simple and did not involve any invasive processes. By transplanting PMSCs into UUO rats, PMSCs were shown to effectively alleviate kidney inflammation by interfering with CD4^+^ T cell polarization. To investigate the underlying mechanism, we further verified how PMSCs affect the secretion of inflammatory factors by CD4^+^ T cells in vitro. However, none of the tested molecules were sufficient to fully account for the function of PMSCs.

MSC-derived extracellular vesicles (EVs) are 100 to 200 nm endosome-derived vesicles [11] and are enriched for a subset of diverse protein and mRNA cargo [12], indicating that they are relatively likely to work as a whole. Many data have also shown that MSC-derived EVs are immunologically active and can induce Treg cells in vitro and in vivo [13]. In this study, we performed further in vivo investigations to explore whether PMSC-EVs can relieve fibrosis and influence the anti-inflammatory switch by modulating the balance of Treg/Th17 cells in renal tissue after UUO.

## 2. Materials and Methods

### 2.1. Animal Modeling and Experimental Methods

Specific pathogen-free (SPF) Sprague-Dawley (SD) rats (male, 8 weeks) and pregnant SD rats (14-16 days) were purchased from the Shanghai Laboratory Animal Research Center. All animals were kept in Shanghai Ninth's Hospital of the Shanghai Laboratory Animal Center. This study was approved by the local ethics committee. All experiments were performed in accordance with the National Institutes of Health Guide for the Care and Use of Laboratory Animals.

Rats were randomly divided into sham and UUO groups, with 18 animals in each group. Operations were performed by exposing the right kidney through a flank incision and then immediately closing the incision (sham) or ligating the ureter immediately distal of the renal pelvis (UUO model) [10]. For in vivo experiments, UUO rats were randomly divided into groups receiving PMSCs intervention (2 × 10^5^ cells) or saline injection as a control via postcava injection at the start of UUO. The rats were sacrificed 3, 7, or 14 days after surgery.

### 2.2. Isolation and Culture of Placenta-Derived Primary Cells

The placenta of SD rats was collected on gestational days 14 to 16, rinsed with sterile PBS [14], cut into small pieces, digested with trypsin and collagenase 6 for 45 mins at 37°C, and then filtered with a 200 *μ*m mesh sieve. After centrifugation at 1,500 rpm, the cells were resuspended in low-glucose DMEM (LG-DMEM) supplemented with 10% heat-inactivated fetal bovine serum. They were then cultured at a density of 4 × 10^5^/ml at 37°C in a 5% CO_2_ atmosphere. The cells were cultured to 80% confluency, and FACS analysis was performed using a FACSCalibur (BD, USA).

### 2.3. Isolation and Characterization of PMSC-EVs

EVs were prepared from the supernatant of PMSC cultures with serum-free LG-DMEM. Briefly, the conditioned medium was collected at 48 h. In total, 170 ml supernatant was processed through 0.45 *μ*m and 0.22 *μ*m filters to remove intact cells and debris. Then, Amicon ultracentrifugal filters (Millipore Sigma, Darmstadt, Germany) were used to concentrate the cell supernatant into 1 ml as a unit dose, and an equal volume of ExoQuick was added and incubated overnight at 4°C. EVs were isolated with a 30-minute low-speed spin (1500 g). The purified EVs were stored at –80°C until use. The expression of the EV markers HSP70, TSG101, CD81, CD9, and CD63 (rabbit monoclonal antibodies (mAbs), Cell Signaling Technology, USA) was detected using Western blotting. The purified EVs were further identified with a nanoparticle tracking analysis (NTA) system and transmission electron microscopy.

### 2.4. Assessment of the Degree of Tubular Injury Using Masson Staining

Paraffin renal tissue sections were stained with Masson's trichrome. A quantitative evaluation based on arbitrary scores was performed by a blinded observer for the indicated histopathological parameters. We evaluated 10 fields (400x) in each pathological section of renal interstitial tissue stained with Masson's trichrome and considered bright blue collagen deposition as a positive signal. Image ProPlus software was used for analysis, and the ratio of the interstitial collagen deposition area to the area without deposition was calculated and averaged.

### 2.5. Immunohistochemical and Immunofluorescence Staining

After deparaffinization, renal sections were treated with 3% H_2_O_2_ for 10 min to inactivate endogenous peroxidases. The sections were blocked with 5% normal rabbit serum and then incubated with primary antibodies specific for *α*-SMA (1 : 100, Abcam), collagen I (1 : 500, Abcam), FoxP3 (1 : 100, Abcam), IL17 (1 : 100, Abcam), and CD4 (1 : 50, Abcam) overnight at 4°C, followed by incubation with secondary antibodies for 60 min. After being washed three times, the tissue sections were visualized by the dropwise addition of peroxidase-labeled streptavidin-biotin (SABC) and 3,3N-diaminobenzidine tetrahydrochloride (DAB) (Dako Corporation).

### 2.6. CD4^+^ T Cell Isolation

CD4^+^ T lymphocytes were obtained from the peripheral blood (PB) of rats and used to establish in vitro groups. Red blood cells were eliminated by density gradient centrifugation, and CD4^+^ T cells were purified (>90%) from the mononuclear cells using CD4^+^ T cell Isolation Kit MicroBeads (Miltenyi Biotec, Bergisch Gladbach, Germany) according to the manufacturer's instructions.

### 2.7. PMSCs and CD4^+^ T Cells Coculture Evaluation

The effect of the addition of PMSCs to CD4^+^ T cell cultures was tested by coculturing cells at a ratio of 10 : 1 CD4^+^ T cells vs. PMSCs. To activate CD4^+^ T cells, purified CD4^+^ T cells were seeded in anti-CD3 Ab (5 *μ*g/ml, BD Biosciences) and anti-CD28 Ab-coated plates (10 *μ*g/ml, BD Biosciences), and treated with IL-2 (10 ng/ml, R&D Systems, USA) for 3 days [15, 16]. Then, a transwell system (Corning, 0.4 *μ*m pore size) was utilized for PMSCs and CD4^+^ T cells coculture. Activated CD4^+^T cells were seeded in the lower chamber while PMSCs were seeded in the upper chamber. After 72 hours incubation, CD4^+^T cells were stained with CD25-APC/Foxp3-FITC or IL17-APC/CD4-PE and analyzed by FCM analysis, respectively. Manufacturer instructions were followed for all procedures.

### 2.8. Flow Cytometry (FCM) Analysis

Rats were sacrificed by cervical dislocation, and the kidneys and spleen were removed under aseptic conditions. Peripheral heparinized blood was collected from the rats. The kidney tissue was digested with collagenase IV for 60 min at 37°C, while the spleen was ground with an injector. They were filtered with a 200 *μ*m mesh sieve, and then single cells were collected and suspended in RPMI 1640 medium. Splenocytes were plated in 24-well plates at a concentration of 1 × 10^6^/ml, and phorbol 12-myristate 13-acetate (PMA), ionomycin, and monensin were added at the concentrations 500 ng/ml, 1 *μ*g/ml, and 1 m*Μ*, respectively (all from Calbiochem, San Diego, CA); the cells were then incubated at 37°C in 5% CO_2_ for 4 h. Red blood cell lysis buffer (4 ml/tube) was then added and incubated for 10 min. After washing, fluorophore-labeled anti-CD4 or anti-CD25 Abs (eBioscience, USA) or an isotype control Ab was added for surface staining for 30 min at room temperature (RT) in the dark, followed by two washes with PBS containing 0.1% bovine serum albumin (BSA). Foxp3 staining procedures were performed according to the manufacturer's instructions. Intracellular staining of T cells was performed after 6 h of activation with PMA (1 ng/ml) and ionomycin (1 mg/ml). Then, the cells underwent surface staining with the fluorochrome-conjugated anti-CD4 Ab. An anti-rat IL-17A PE-conjugated Ab (2 *μ*l) in 100 *μ*l staining buffer was added to the cell pellet after centrifugation and incubated at 4°C for 30 min in the dark. Flow cytometry analysis of cytokine production was performed using an anti-IL-17 allophycocyanin-conjugated mAb.

PMSCs were incubated for 20 min at 4°C with combinations of the following Abs conjugated to fluorescein isothiocyanate (FITC) or Phycoerythrin (PE): anti-CD45 (BioLegend), anti-CD90 (eBioscience), anti-CD34 (BioLegend), anti-CD105 (eBioscience), anti-HLA (BioLegend), and anti-CD29 (eBioscience).

### 2.9. ELISA Analysis of Cytokine Production

Serum was collected from rat PB, and the supernatant of a renal cortex homogenate generated from right kidney tissue pestled in a homogenizer was also collected. In vitro, each well of a culture plate suspension was centrifuged, and the cells in the pellet were collected. The supernatant was used for enzyme-linked immunosorbent assay (ELISA) (eBioscience) analysis of inflammatory factors.

## 3. Results

### 3.1. Characterization of PMSCs and PMSC-EVs

**Figure 1:**
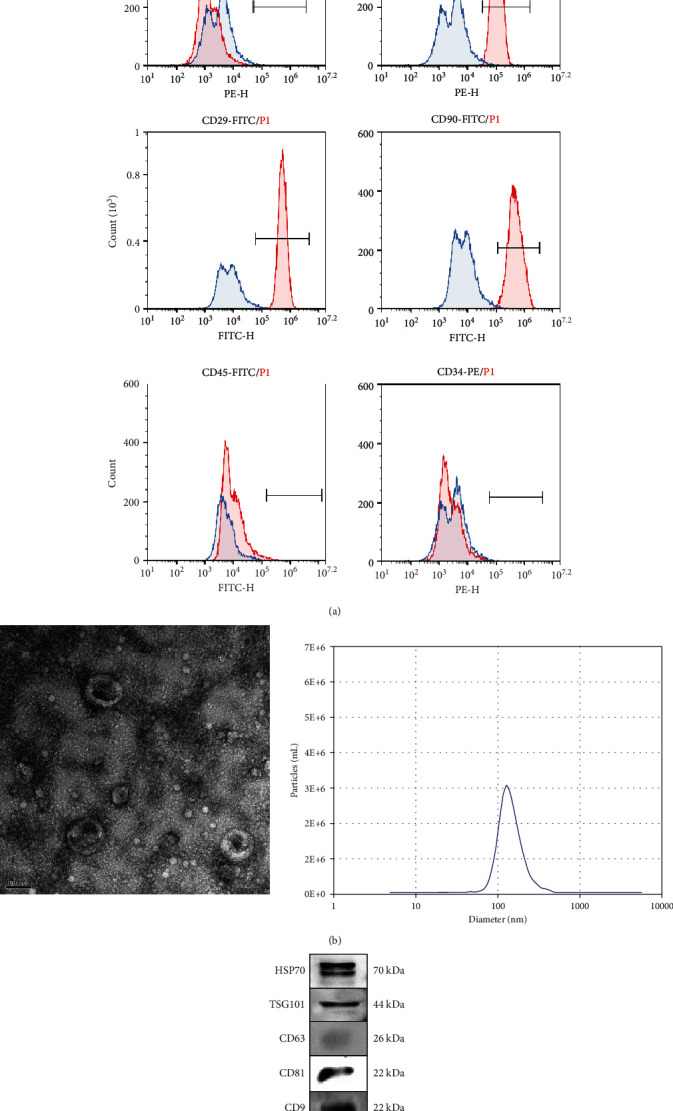
Characterization of PMSC and PMSC EVs. (a) PMSC phenotypes were tested by flow cytometry. PMSCs were trypsinized and stained with MSC-related markers CD29, CD90, and CD105 and hematopoietic markers CD45, CD34, and HLA. (b) PMSC-EVs were extracted from cell supernatant. Representative micrograph of purified PMSC-EVs under transmission electron microscopy showing a cup-shaped membrane vesicle 30-100 nm in diameter (30,000×). Purified PMSC EVs particles have a diameter of 100 nm. (c). Expression levels of typical molecular markers of EVs were tested by Western blot including HSP70, TSG101, CD63, CD81, and CD9.

PMSCs from pregnant rats were successfully isolated by adherence separation and reached 80-90% confluence by day 14 with a stable “fibroblast-like” spindle morphology. The MSC profile was confirmed based on positivity for the MSC-related markers CD29, CD90, and CD105 in the absence of positive for the hematopoietic marker CD45 CD34 and HLA, and the profile was evaluated by flow cytometry (Figure 1(a)). As indicated by our previous research, PMSCs are able to differentiate into adipocytes, chondrocytes, and osteoblasts under the appropriate culture conditions [10].

EVs were extracted from PMSCs, purified, and identified by morphology and EV marker expression An electron micrograph of phosphotungstic acid-stained EVs is shown (Figure 1(b)). The particle size and concentration of EVs were measured with an NTA system, with particle size peaking at 110 nm diameter (Figure 1(b)). Purified PMSC-EVs expressed EV marker proteins such as HSP70, TSG101, CD81, CD9, and CD63 (Figure 1(c)).

### 3.2. PMSCs Attenuate Renal Interstitial Fibrosis in a UUO Model

**Figure 2:**
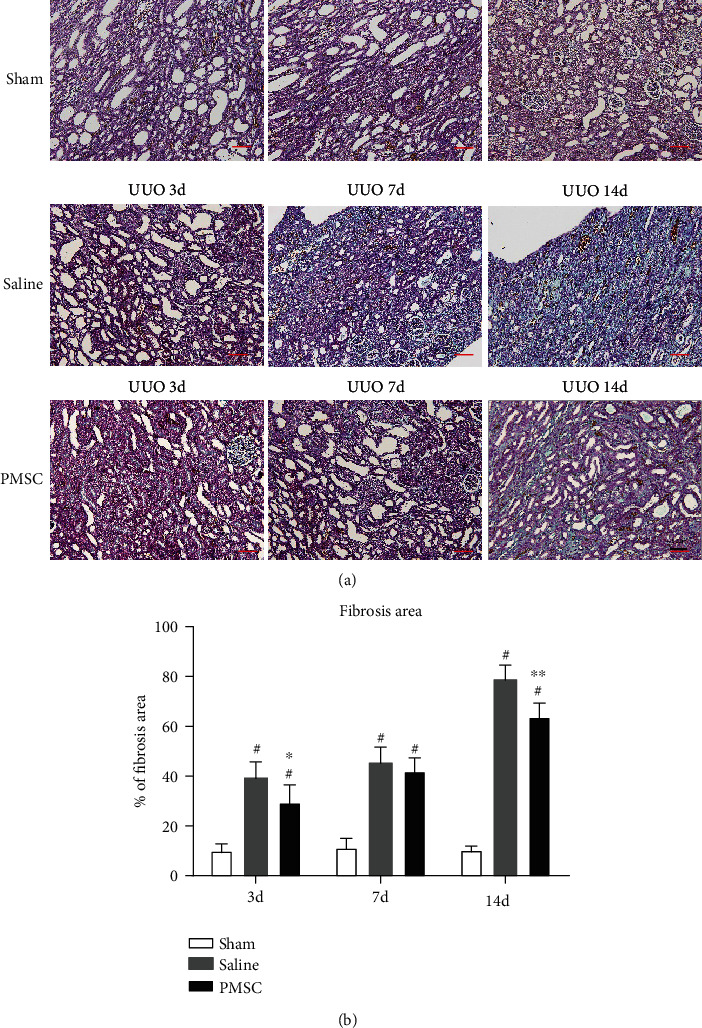
PMSCs regulate the UUO rat renal tubulointerstitial fibrosis. (a) Masson's trichrome staining of kidney sections from normal, saline, and PMSCs treated rats at day 3, 7, and 14 after UUO. Sham group (*n* = 5) represents the kidney from untreated rats after 3, 7, and 14 days, respectively. Saline group (*n* = 5) represents saline-treated kidney subjected to UUO after 3, 7, and 14 days, respectively. PMSC group (*n* = 5) represents PMSCs treated kidney at 3, 7, and 14 days, respectively. Scale bar: 50 *μ*m. (b) Tubulointerstitial infiltration was quantified by image analysis using NIS Elements (Nikon). ^∗^*P* < 0.05 compared with Saline group; ^∗∗^*P* < 0.01 compared with Saline group; ^#^*P* < 0.05 compared with Sham group.

Masson's trichrome staining demonstrated that collagen accumulation in the interstitium progressively increased in the UUO group, and there was a significant reduction in the fibrotic area in the MSC-treated group (*P* < 0.05; Figure 2(a) and 2(b)). Masson staining quantification showed that fibrosis was significantly reduced in the PMSC-treated group compared with the 3-day (*P* < 0.05) and 14-day UUO groups (*P* < 0.01).

### 3.3. PMSCs Intravenous Transplantation Promoted Treg Cell Infiltration into Obstructed Kidneys

**Figure 3:**
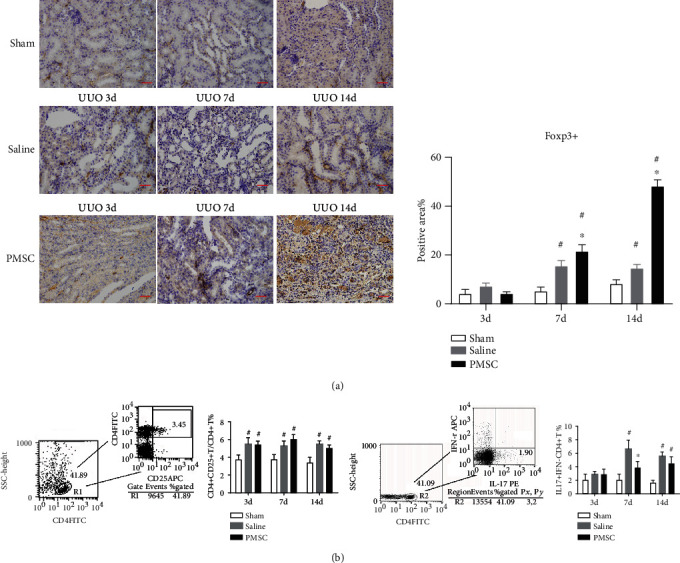
Effects of PMSCs injection on regulating CD4^+^CD25^+^Foxp3 T cells and IL17^+^IFN^−^T cells of UUO rats in vivo. (a) IHC staining of Foxp3 in kidney sections from Sham, Saline, and PMSC group at days 3, 7, and 14 after UUO, respectively. Representative pictures are acquired. Scale bar: 50 *μ*m. Foxp3 positive percentage was quantitated by measuring positive area from representative images and analyzed by image J software. ^∗^*P* < 0.05 compared with Saline group; ^∗^*P* < 0.01 compared with Saline group; ^#^*P* < 0.05 compared with Sham group. (b) The percentage of CD4^+^CD25^+^ T cells in CD4^+^ T cells extracted from PMNCs as well as the percentage of IL-17^+^IFN^−^*γ* T cell in CD4^+^ T cells extracted from rat spleen homogenate was analyzed by FACs, respectively. ^#^ indicates *P* < 0.05 VS. sham. ^∗^*P* < 0.05 compared with Saline group; ^#^*P* < 0.05 compared with Sham group.

Immunohistochemical staining (Figure 3(a)) showed that Foxp3^+^ positive lymphocytes were mainly located in the foci of tubulointerstitial cells. After the UUO model establishment, renal interstitial infiltration of Foxp3^+^ Treg cells was significantly higher in the PMSC intervention group than in the other groups on day 14. Flow cytometry analysis indicated that the PB CD4^+^CD25^+^ Treg/CD4^+^ cell ratios in the PMSC intervention group and saline injection group were significantly higher than the ratio in the sham group (*P* < 0.05). Moreover, there was no statistically significant difference between the PMSC intervention group and the saline injection group (*P* > 0.05) (Figure 3(b)).

Flow cytometry results also showed that in the rat spleen at 7 and 14 days after UUO model establishment, the percentage of IFN^−^IL17^+^CD4^+^ T cells in the CD4^+^ T cell population in the UUO group was significantly higher than that in the sham group (*P* < 0.05, Figure 3(b)). However, there was no statistically significant difference between the saline injection and PMSC intervention groups 3 days after UUO modeling (*P* > 0.05, Figure 3(b)).

### 3.4. PMSCs Affect the Treg/Th17 Cell Balance among Total CD4^+^ T Cells In Vitro

**Figure 4:**
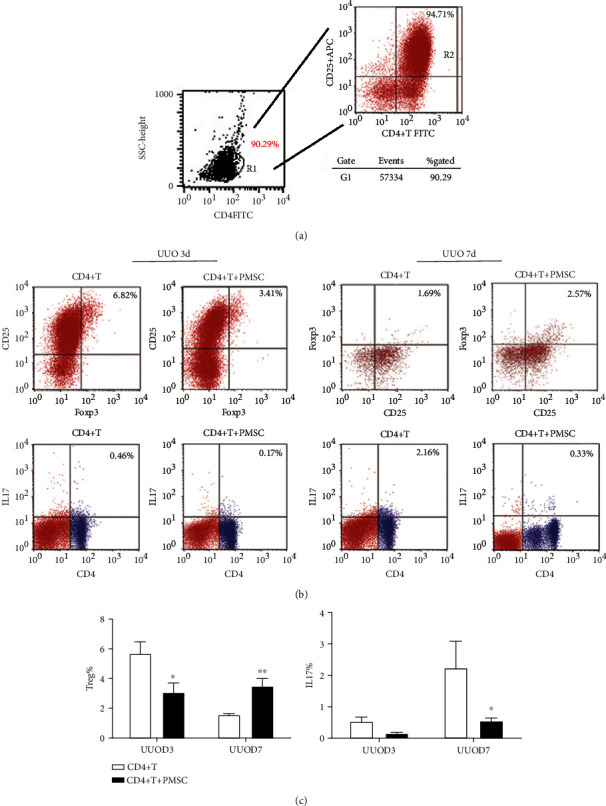
PMSCs treatment increase Treg and decrease Th17 expression in CD4^+^ T cells from PBMC in vitro. (a) CD4^+^T cells were obtained from PBMC of rats at day 3 and 7 after UUO via microbeads, respectively, and cocultured with or without PBMCs. (b) A 0.4 *μ*m transwell system was used and activated CD4^+^T cells were seeded in the lower chamber while PMSCs were seeded in the upper chamber. After 72 hours of incubation, CD4^+^T cells were stained with CD25-APC and Foxp3-FITC or IL17-APC, CD4-PE, respectively. (c) Histograms representation of the percentage of Treg cells (CD25^+^ Foxp3^+^) and IL17^+^ CD4^+^T cells in CD4^+^T cells cocultured with or without PMSC. Error bars are ±SD. ^∗^*P* < 0.05 compared with CD4^+^T cells group; ^∗∗^*P* < 0.01 compared with CD4^+^T cells group. *n* = 4 in each group.

To further analyze the effect of PMSCs on the profiles of CD4^+^ T cells, we evaluated the Treg/Th17 cell balance among total CD4^+^ T cells with or without the addition of PMSCs after 3 days of activation. With PMSC coculture, the production of Treg cells and IL-17 by CD4^+^ T cells cultured under baseline stimulation remarkably promoted CD4^+^ T proliferation and differentiation (Figure 4(a)). Furthermore, in these cultures, the addition of PMSCs markedly suppressed the expansion of Th17 cells 3 days after UUO modeling (*P* < 0.05; Figures 4(b) and 4(c)) and upregulated the level of CD4^+^CD25^+^FOXP3^+^ Treg cells at 7 days after UUO modeling (*P* < 0.05) (Figures 4(b) and 4(c)).

### 3.5. Influence of PMSCs on Inflammatory Cytokine Production by CD4^+^ T Cells through a Paracrine Effect

**Figure 5:**
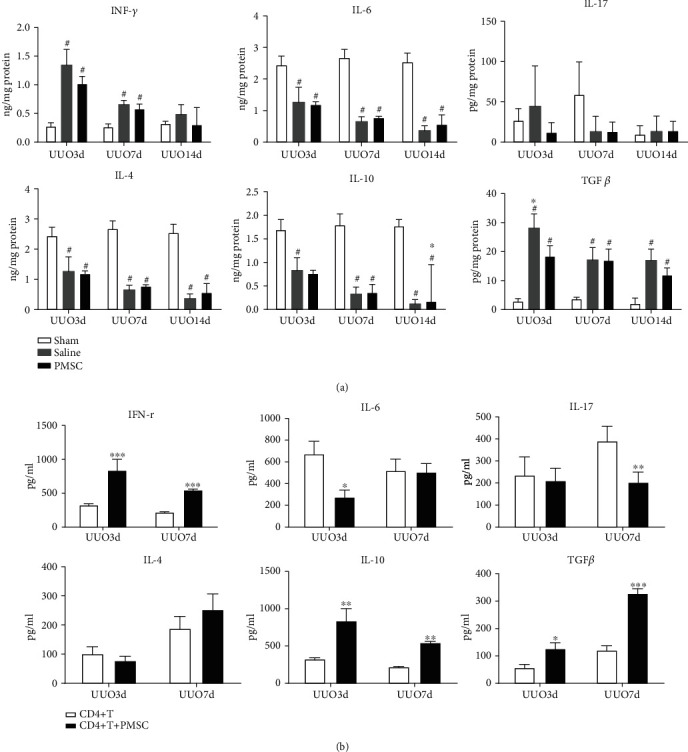
PMSCs regulate the expression of inflammatory cytokines including IFN-*γ*, IL-4, IL-6, IL10, IL-17, and TGF-*β* in vivo and vitro. (a) ELISA analysis was performed to detect cytokines expression both from renal tissue homogenate and (b) in vitro experiments. (b) Inflammatory factors tested from cell supernatant of CD4^+^T cells extracted from PMNC of rats at days 3 and 7 after UUO with or without PMSC coculture (*n* = 4). ^∗^*P* < 0.05 compared with CD4^+^T cells group; ^∗∗^*P* < 0.01 compared with CD4^+^T cells group. ^∗∗∗^*P* < 0.001 compared with the CD4^+^T cells group.

We analyzed the effects of PMSCs on the profiles of CD4^+^ T cells both in vivo and in vitro. We detected the levels of the proinflammatory cytokines IFN-*γ*, IL-6, and IL-17, and we also tested the expression of the anti-inflammatory cytokines IL-4 and IL10. In the in vivo experiment, we found that the levels of IFN-*γ* were significantly increased but the expression of IL-4, IL-6, and IL-10 was decreased significantly on days 3 and 7 in the UUO group compared with the sham operation group. IL-10 was the only cytokine upregulated in response to PMSC intervention on the 14^th^ day after UUO modeling (*P* < 0.05) (Figure 5(a)). For other cytokines, no significant differences were observed between the PMSC treatment group and the saline injection group (*P* > 0.05).

In in vitro experiments, we tested cytokines in each group culture plate suspension. Our data showed that the levels of IFN-*γ* were significantly decreased when CD4^+^ T cells were cocultured with PMSCs on both day 3 and day 7 after UUO modeling (*P* < 0.05). The expression of IL-6 decreased on day 3, and that of IL-17 decreased on day 7 in the PMSC coculture group (*P* < 0.05). The levels of TGF-*β* and IL-10 were significantly increased in the PMSC coculture group on both day 3 and day 7 (*P* < 0.05). There were no significant differences in IL-4 expression between the PMSC coculture group and the CD4^+^ T cell group (Figure 5(b)).

### 3.6. PMSC-EVs Alleviate Renal Tubulointerstitial Fibrosis and Affect Foxp3+/IL-17^+^ Cell Infiltration into the Kidneys of UUO Rats on Day 7

**Figure 6:**
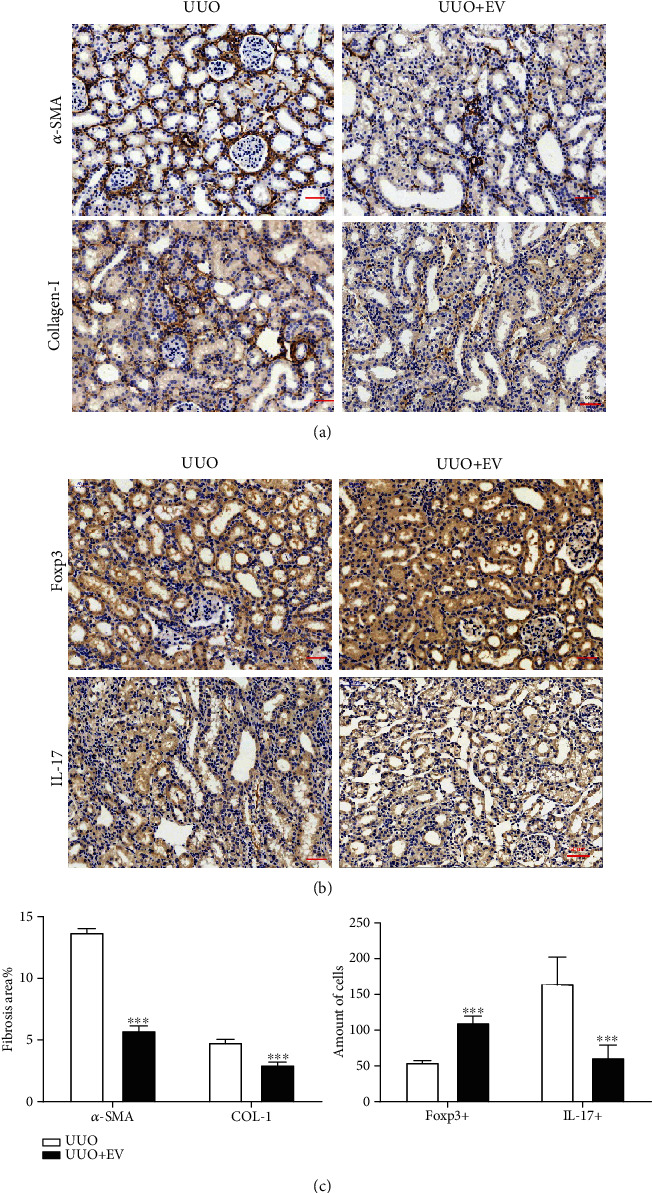
PMSC-EVs treatment attenuate tubulointerstitial fibrosis and increase Foxp3 expression by downregulating IL17 expression in vivo. (a) IHC for detecting *α*-SMA and COL-I expression in rat renal tissues from the UUO group and UUO+EV treatment group. Scale bar: 50 *μ*m. (b) IHC for Foxp3 and IL-17 in rat renal tissue sections 7 days after UUO with or without PMSC-EVs treatment. Scale bar: 50 *μ*m. (c) Fibrosis area and Foxp3 and IL17 positive cells in representative images from (a) and (b) were analyzed by Image J software. Error bars are ±SD. ^∗^*P* < 0.05 compared with the UUO group; ^∗∗^*P* < 0.01 compared with the UUO group. ^∗∗∗^*P* < 0.001 compared with UUO group. (d) IF for identification of Foxp3^+^ T cells infiltration in renal tissue after UUO 7 days with or without PMSC-EVs treatment. Scale bar: 50 *μ*m. (e) Representative IF staining pictures of IL-17^+^ T cells in the UUO group and UUO+EV group. Scale bar: 50 *μ*m.

EV-treated rats showed less collagen deposition in the renal interstitium than untreated rats, as quantified by *α*-SMA staining and immunohistochemical staining (Figures 6(a) and 6(c)). In addition, the number of CD4^+^ T cells infiltrating the affected kidneys was reduced in the EV-treated group compared with the untreated group (Figures 6(b)–6(e)).

## 4. Discussion

In the UUO model, interstitial inflammatory cell infiltration progressively increased from 12 h after obstruction through up to 14 days. The infiltrated leukocytes were mainly macrophages and T lymphocytes. The role of T lymphocytes in the progression of renal fibrosis was significantly highlighted. Depletion of CD4^+^ T cells in wild-type mice with a mAb significantly reduced the amount of interstitial expansion and collagen deposition after 2 weeks of obstruction [17]. CD4^+^ T cells can differentiate into three subpopulations, the Th1, Th2, and Th17 subsets [18]. The pivotal role of Th2 cells versus the classic role of Th1 cells was recently highlighted, and the Th17 response was also enhanced in the UUO model [19–21]. These studies indicated that renal interstitial injury could be improved by interfering with the polarization of CD4^+^ T cells.

In previous research, the effectiveness of bone marrow stem cell (BMSC) therapy on UUO rats was apparent, and we found that BMSCs could quantifiably ameliorate fibrosis. In this study, there was no significant relief in fibrosis after PMSC treatment. This may be related to the insufficient quantities of stromal cells, and some of these cells were damaged by phagocytosis in the pulmonary vascular system.

Moreover, we found that the ratio of CD4^+^CD25^+^ T cells to CD4^+^ T cells in the PB and the ratio of IL17A^+^IFN*γ*^−^CD4^+^ T cells to CD4^+^ T cells in the spleen were significantly higher in the UUO group than in the control group. These results suggest that Treg and Th17 cells are involved in the processes of renal interstitial inflammation and fibrosis. In addition, Foxp3 expression in kidney tissue sections from the PMSC intervention group was significantly higher than that in tissue sections from the other groups at 1 or 2 weeks after UUO establishment. These results indicate that PMSCs may relieve tissue injury by increasing the infiltration of Treg cells. T cells that express the transcription factor Foxp3 are a regulatory subset of CD4^+^ Th cells, which are necessary for the maintenance of peripheral tolerance and immune homeostasis. Recent studies have suggested that Foxp3^+^ Treg cells regulate both Th1- and Th17-mediated effector responses [18, 22]. Thus, Foxp3^+^ Treg cells may help to suppress pathogenic inflammation.

MSCs exert immunomodulatory effects not only via direct cell-cell contact but also by releasing soluble factors such as IL-10 and TGF*β* [23, 24]. The varied expression of these factors will lead to differences in regulating immunosuppressive effects. IFN-*γ*, which is a Th1 cell-related factor, is a proinflammatory factor and a crucial effector molecule in immunological rejection [25]. In contrast, IL-10 is regarded as a Th2-related anti-inflammatory factor, like IL-4, and plays a key role in the induction of Treg cells [26]. It also recruits Treg cells that are responsible for the regulation of the immune system. In this study, we found that IL-10 could be secreted by PMSCs, which may synergistically relieve inflammatory responses.

After UUO modeling, the expression levels of TGF-*β*, IFN-*γ*, and IL-6 were upregulated, while the levels of IL-4 and IL-10 were downregulated. Fourteen days after the model establishment, the PMSC intervention group showed a higher level of IL-10 than the saline infusion group. These data suggest that PMSCs may influence the conversion of the inflammatory environment into an anti-inflammatory environment by upregulating the expression of the anti-inflammatory factor IL-10 and increasing Foxp3 expression locally in renal tissue, which ultimately leads to renal protection.

To further investigate the effects of PMSCs on CD4^+^ T cells in vitro, we isolated PB mononuclear cells from UUO rats. PMSCs were cocultured with CD4^+^ T cells from UUO rats. PMSCs increased the proportion of CD4^+^CD25^+^Foxp3^+^ Treg cells in the total CD4^+^ T cell population, whereas the ratio of IL17A^+^CD4^+^ T cells to CD4^+^ T cells was significantly lower in the coculture group than in the purified CD4^+^ T cell group. Luz-Crawford et al. [27] also found that MSCs were able to suppress the proliferation, activation, and differentiation of CD4^+^ T cells induced to differentiate into Th1 and Th17 cells. We also found that the secretion of anti-inflammatory cytokines such as IL-6 and IL-10 was significantly increased in purified CD4^+^ T cells isolated from UUO rats. However, the secretion of IFN-*γ*, which is considered a proinflammatory cytokine, was decreased in the PMSC coculture group, and the expression level of IL-17 was lower in the PMSC coculture group than in the CD4^+^ T cell group. This study demonstrates that MSCs contribute to the generation of an immunosuppressive environment via the inhibition of proinflammatory T cells and the induction of T cells with a regulatory phenotype.

Previous studies have shown that MSCs secrete immunologically active EVs, which can enhance the secretion of anti-inflammatory cytokines [28]. MSC-derived EVs enhance Treg production in vitro and in vivo through an antigen-presenting cell- (APC-) mediated pathway by transferring RNA, proteins, and bioactive lipids [13]. In our study, we injected PMSC-EVs that reached the target directly to improve immunoregulatory efficiency. We found that PMSC-EVs had an effect similar to that of MSCs in regulating the Treg/Th17 cell balance in renal tissues after UUO establishment. All the evidence may show that PMSCs regulate immunocytes by changing the subtypes of CD4^+^ T cells by a paracrine mechanism.

In conclusion, this study reveals that PMSCs and PMSC-EVs suppress the proliferation of CD4^+^ T cells and induce CD4^+^ T cells to differentiate into Treg and Th2 cells but not Th17 and Th1 cells. PMSCs and PMSC-EVs may convert the inflammatory environment into an anti-inflammatory environment by releasing paracrine factors, which ultimately leads to renal protection under UUO conditions. These findings provide a basic foundation for the future use of EVs as a new biological therapeutic for renal injury or immunological disease.

## Data Availability

The data used to support the findings of this study are available from the corresponding author upon request.
